# An Aggressive Central Giant Cell Granuloma of Mandible in an Older Patient Managed Successfully With Marginal Mandibulectomy and Reconstruction With Submental Island Flap

**DOI:** 10.7759/cureus.15414

**Published:** 2021-06-03

**Authors:** Sumit Anand, Arunkumar KV

**Affiliations:** 1 Oral and Maxillofacial Surgery, Teerthanker Mahaveer Dental College and Research Centre, Teerthanker Mahaveer Hospital, Teerthanker Mahaveer University, Moradabad, IND

**Keywords:** multinucleated giant cells, central giant cell granuloma, marginal mandibulectomy, reconstruction, sub mental island flap

## Abstract

The Central giant cell granuloma (CGCG) is a non-odontogenic, osteolytic lesion of unknown aetiology, which affects the craniofacial region, particularly the anterior mandible. The age group commonly affected is below 30 years, with a distinct female predilection. Histopathological analyses show fibro cellular stroma consisting of evenly distributed multinucleated giant cells, multiple foci of haemorrhage, and focal areas of spicules of newly formed bone. Depending upon the extent, behaviour, and characteristics management varies from non-surgical to surgical approaches. Since CGCG is associated with a higher rate of recurrence, excision by curettage with the removal of peripheral bone margins is the gold standard and radical surgical intervention in aggressive lesions is associated with low recurrences. Reconstruction of the resulting surgical defect is extremely important to restore aesthetics and function. This case report reviews presentation along with currently used therapies for CGCG while describing an uncommon case of locally aggressive CGCG occurring in a 50-year-old female involving the posterior mandibular region, successfully managed with marginal mandibulectomy, curettage and reconstructed with submental island flap with no recurrence during follow up.

## Introduction

The central giant cell granuloma (CGCG) is a non-odontogenic, osteolytic lesion of unknown aetiology, which affects the craniofacial region, particularly the anterior mandible. It is histologically benign, although locally proliferative and destructive from osteoclastic activity [1]. It was first described by Jaffe in 1953 and termed “giant cell reparative granuloma”. The term “reparative” was later dropped due to subsequent experiences revealing inconsistencies between clinical behaviour of the lesion and a reparative process [2]. World Health Organization, defines CGCG as “an intraosseous lesion consisting of cellular fibrous tissue containing multiple foci of hemorrhage, aggregations of multinucleated giant cells (MNGC), and occasionally, trabeculae of woven bone” [2]. CGCG accounts for less than seven per cent of all benign jaw tumours [3]. The mandible is more commonly affected than the maxilla, with a comparative ratio varying from 2:1 to 11:9. It is typically seen in the mandibular body area, anterior to the first molar. The age group most commonly affected is below 30 years, with a distinct predilection for females [4]. Radiographically it appears as solitary, unilocular, or multilocular radiolucencies. Clinical behaviour of a CGCG varies from an indolent slowly growing asymptomatic lesion to aggressively rapid bone hollowing with cortical expansion, thinning and perforation, root resorption, displacement of teeth and nerves, along with featuring pain [5]. Depending upon the extent, behaviour, and characteristics management varies from non-surgical to surgical approaches. CGCG is often associated with a higher rate of recurrence (11% to 49% and up to 72% for aggressive lesions) [6]. Excision by curettage with the removal of peripheral bone margins is the gold standard and a radical surgical intervention in aggressive lesions is associated with low recurrence rates. Even though such a radical procedure is effective, an inevitable loss of teeth will result, along with disturbance of the inferior alveolar nerve function [6]. Reconstruction of the resulting surgical defect is extremely important to restore aesthetics as well as function. This article reviews presentation and currently used therapies for CGCG while describing an uncommon case of locally aggressive CGCG occurring in a 50-year-old female involving the posterior mandibular region.

## Case presentation

**Figure 1:**
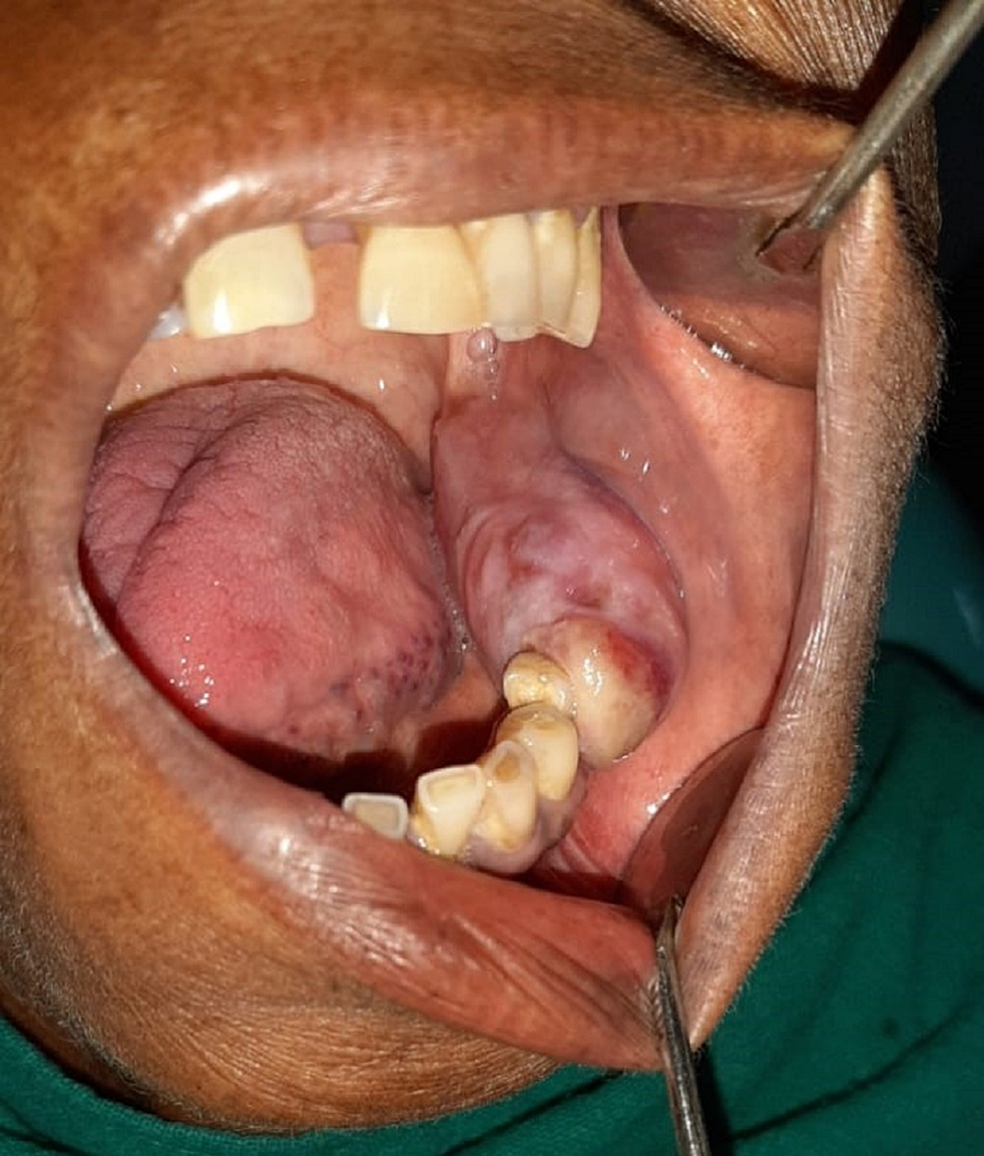
Pre-operative clinical picture showing growth over the lower left alveolar region.

**Figure 2:**
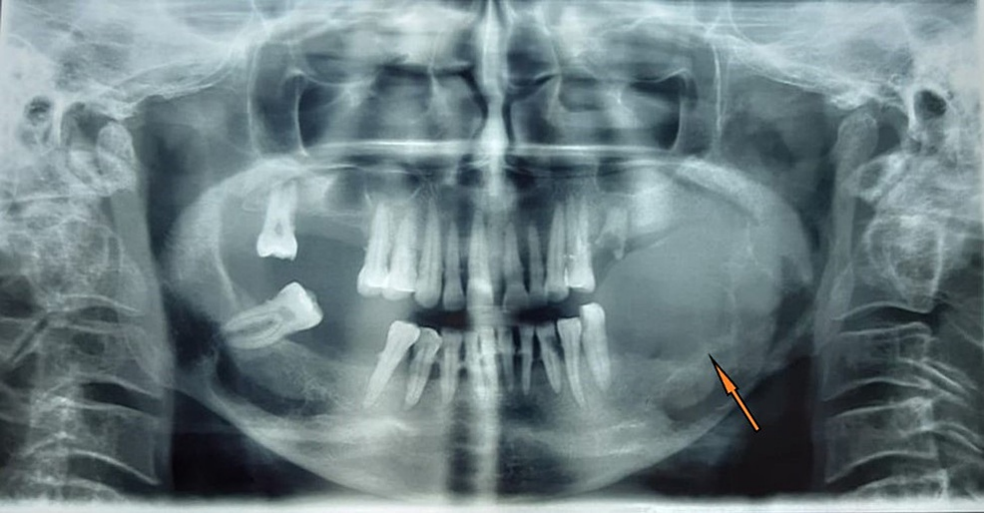
Orthopantomogram showing a well-defined radiolucent lesion extending from the lower left first premolar to retromolar region.

**Figure 3:**
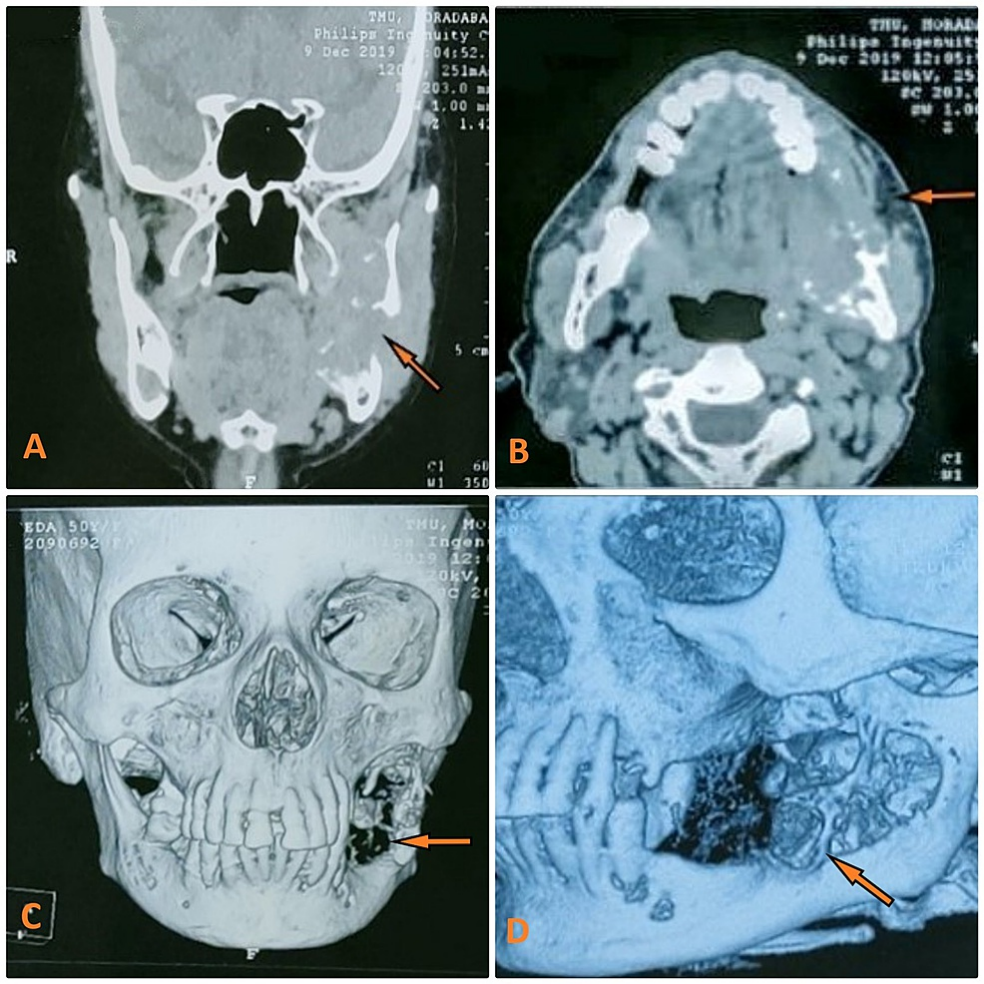
Computed tomography scans revealing aggressive bony resorption of the lower left jaw starting from premolar region and extending well into the mandibular ramus. (A) Coronal view. (B) Axial view. (C) Three-dimensional reconstruction frontal view. (D) Three-dimensional reconstruction lateral view.

**Figure 4:**
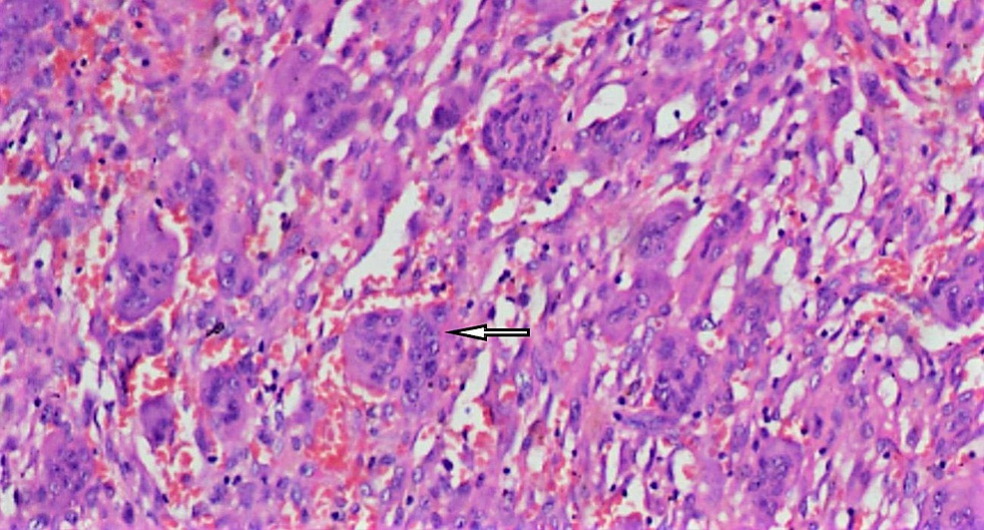
Histopathological analyses revealing fibro cellular stroma consisting of multinucleated giant cells distributed evenly throughout the stroma.

A 50 years old female patient reported to our department in December 2019 and presented with intraoral swelling at the left mandible for six months (Figure 1). Two months after the initial observation of swelling by the patient, she went for extraction of lower left back teeth, which resulted in the swelling growing more rapidly. She also reported pain in the area of the lesion; however, there was no history of any fever or difficulty in swallowing. Medical history was non-significant. On extraoral examination, facial asymmetry was noted in the lower left facial region; however, there was no evidence of any regional lymphadenopathy. Intraoral clinical evaluation revealed linguoverted mandibular premolars along with expansile mass with anteroposterior as well as buccolingual expansion extending from the region of the first premolar up to retromolar area, measuring approximately 6×4 cm (Figure 1). The overlying mucosal colour and texture appeared normal, and on palpation, the lesion was firm with moderately defined borders. The radiographic orthopantomogram revealed a well-defined radiolucent lesion extending from the lower left first premolar to retromolar area (Figure 2). Computed tomography (CT) scans confirmed the presence of aggressive bony resorption of the lower left jaw starting from the premolar region and extending well into the mandibular ramus (Figures 3A-3D). CGCG, ameloblastoma, odontogenic myxoma, traumatic bone cyst, aneurysmal bone cyst, and brown tumour were considered differential diagnosis. Histopathological analysis of a biopsy specimen confirmed an aggressive CGCG (Figure 4). Brown tumour of hyperparathyroidism was ruled out based on patient’s blood profile observations: alkaline phosphatase 107 IU/L (normal range 40 to 140); calcium 10.1 mg/dL (normal range 8.6 to 10.3); phosphate 3.9 mg/dL (range 2.5 to 4.5); parathromone 51 pg/mL (normal range 14 to 65).

**Figure 5:**
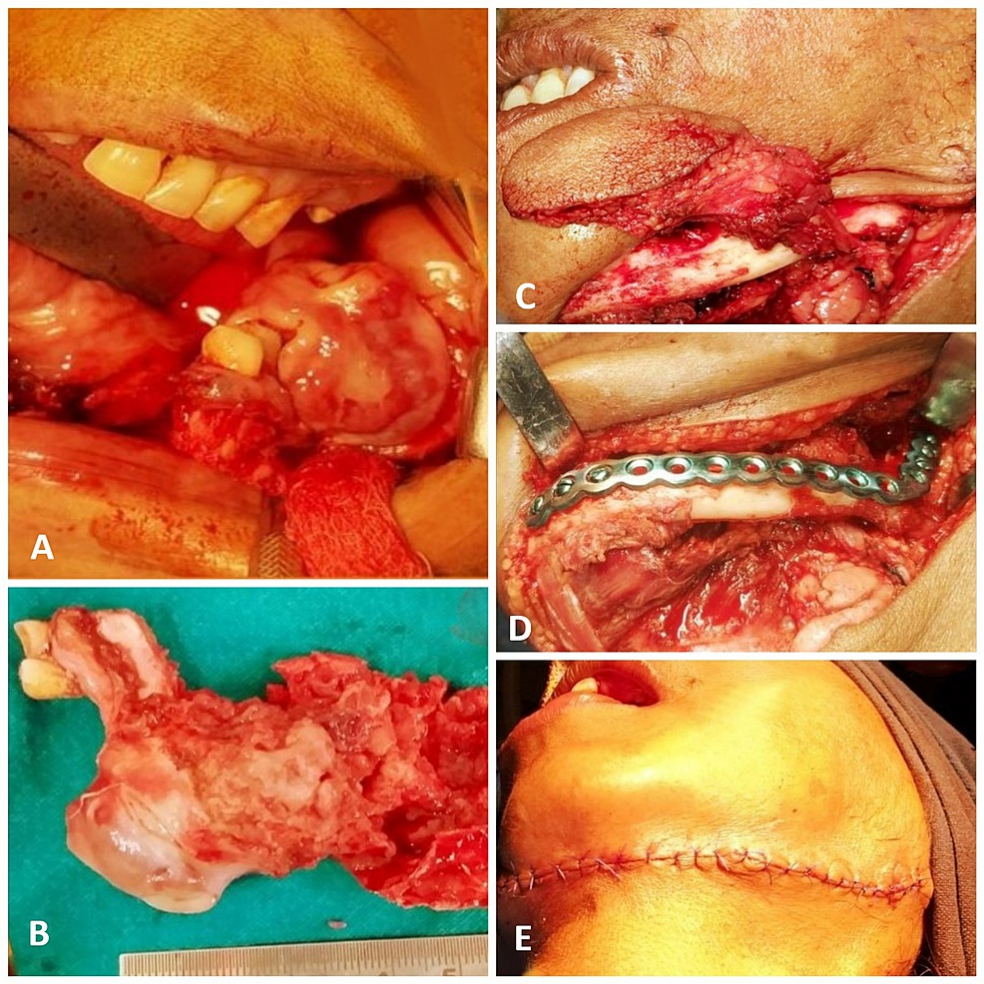
Intra-operative pictures. (A) Lesion being excised. (B) Excised lesion. (C) Submental flap raised and mobilized. (D) Flap transported intraorally to fill the defect and titanium plate fixed to mandible for strength. (E) Closure of donor site.

**Figure 6:**
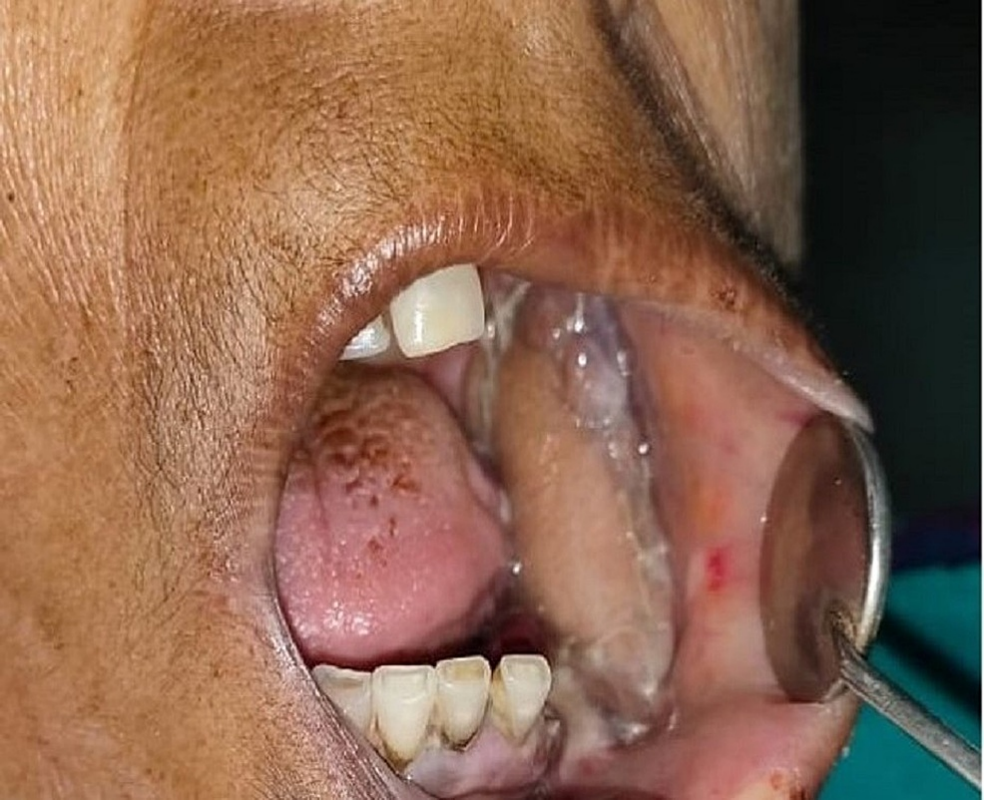
One week post-operative clinical picture showing a healthy flap in the reconstructed region.

Considering the aggressive nature of the lesion as well as the patient’s age, the treatment plan included surgical excision and reconstruction using a submental island flap, under general anaesthesia (GA). Surgical Procedure: A combination of extraoral and intraoral approach was used to excise the lesion completely. Extended marginal mandibulectomy and bone debridement were done to obtain adequate safety margins (Figures 5A, 5B). For reconstruction submental island flap was used. Submental skin was pinched to determine antero-posterior dimension of the flap. Flap design was marked measuring approximately 7 × 5 cm. Following the submental island flap design incision was placed below the lower border of the mandible taking care of the marginal mandibular nerve, and extended through the platysma avoiding injury to common and anterior facial veins. Subplatysmal flap was raised to reveal the facial artery appearing above postero-superior edge of the submandibular salivary gland. The submental artery was then identified at its origin from the facial artery just below the mandible. Similarly, the submental vein was identified at its entry into the facial vein. Dissection was done anteriorly along the submental artery and vein. Thereafter branches and tributaries between the submental artery and vein and the submandibular salivary gland were divided and ligated. A cuff of soft tissue was preserved around the pedicle in order to avoid any vascular compromise. Skin, subcutaneous fat, and platysma were incised around the perimeter of the flap followed by mylohyoid and contralateral digastric muscle. Then anterior belly of the digastric was divided at its attachment on the mandible. The island flap was then raised and mobilized (Figure 5C). The large excised defect was then reconstructed with a pedicle submental flap, which was inset to cover the defect (Figure 5D). To avoid any pathological fracture as a result of a weakened lower jaw, it was supported using a titanium reconstruction plate (Figure 5D). The donor site was closed primarily (Figure 5E). There was satisfactory post-operative wound healing, with good recovery of the patient (Figure 6). In subsequent follow-ups, till one year, no evidence of recurrence was observed clinically as well as radiographically. The donor site healed uneventfully with minimal scars, which was present away from aesthetically visible regions of the face.

## Discussion

The CGCG has two major cell types in its highly cellular fibroblastic stroma: Spindle-shaped cells and MNGC. Out of these two cell types the spindle-shaped cells contribute to tumour proliferation. Even though precise aetiology is unclear, it has been hypothesized that due to some epigenetic events the spindle-shaped cells gets transformed into tumour cells. These tumour cells later release cytokines which mediate the recruitment of monocytes from blood and transform them into osteoclast-like cells (MNGC). MNGC then cause bone resorption and local proliferation of lesion [7]. Various bone tumours like the CGCG, aneurysmal bone cyst, ameloblastoma, ossifying fibroma, odontogenic myxoma, sarcomas, and arteriovenous malformations might present with apparently similar radiographic appearance [5]. Non-Hodgkin's lymphoma may also on rare instances manifest intraorally [8]. In this case, the histopathological analyses of the biopsy specimen revealed fibro cellular stroma consisting of MNGC distributed evenly throughout the stroma (Figure 4). The giant cells consisted of six to seven small and oval nuclei having prominent nucleoli. Focal areas showed merging of giant cells to forms larger cells. The stroma was composed of thick and thin collagen fibre bundles containing oval to spindle connective tissue cells along with focal areas of spicules of newly formed bone. Numerous endothelial cells lined blood vessels were seen in the stroma along with focal areas of haemorrhage. These findings along with bone resorption seen on the CT scan led to the decisive diagnosis of CGCG.

Non-surgical therapy using pharmacological agents have been reported in the literature by various authors: Jacoway et al. first proposed intralesional steroid injections for CGCG [9]. The hypothesis for steroid use is that extracellular production of lysosomal proteases by giant cells is restricted thereby interfering with the bone resorption process. Also, steroids tend to induce apoptosis of osteoclast-like cells [4,6]. Abdo et al. suggested an intralesional injection of triamcinolone (10 mg/mL) and a local anaesthetic (Marcaine 0.5% with Epinephrine 1:200,000) for a duration of six weeks [10]. Ferretti et al. suggested biweekly intralesional steroid injections for four weeks [11]. However, the disadvantage of steroid therapy is the risk of adrenal suppression. Tarsitano et al. suggested a subcutaneous injection of 3 × 106 IU Interferon alpha 2a /day [12]. Schreuder et al. proposed Calcitonin (100IU/day) subcutaneous injections or nasal sprays as a mode of conservative treatment [13]. Calcitonin promotes osteoblastic activity and halts bone resorption. These are however not much cost-effective. Human monoclonal antibodies such as denosumab have shown good results in the management of CGCG. These antibodies inhibit the maturation process of osteoclasts by binding to receptor activator of nuclear factor-kappa. However, these antibodies carry a risk of medicine-related osteonecrosis of the jaw (MRONJ). Bredell et al. proposed a loading dose of subcutaneous 120 mg with an additional 120 mg on day 8 and day 15 followed by every four weeks for a duration of a minimum of 12 months [14].

Surgical therapy is the gold standard for the treatment of CGCG. The most commonly used intervention is curettage. It can range from a simple curettage to resection. Additionally, combination with cryosurgery and peripheral ostectomy helps to reduce the rate of recurrence. The lowest recurrence has been reported with en bloc surgical resection with a 5-mm margin [8]. Subsequently incorporating platelet-rich fibrin can speed up the filling and re-ossification of the bony defect. PRF can be a rich autologous source of growth factors delivered in high concentrations to the site of bone defect or a region requiring augmentation [15]. In the present case, the nature of the lesion was aggressive and the size was considerably large. Therefore, we performed extended marginal mandibulectomy and bone debridement followed by reconstruction with submental island flap, which resulted in satisfactory recurrence-free outcome in post-operative follow-ups. Submental island flap has emerged as a reliable option in head and neck reconstructions [16]. It provides a relatively adequate, easy to harvest, and well-vascularized tissue, which eliminates the need for a second-stage operation of flap division or resource-intensive and technically challenging microvascular free tissue transfers, thereby resulting in shorter operative time. The additional advantage is that its donor site defect can be closed primarily most of the time because of the laxity of the submental skin, especially in elderly patients [17]. In elderly patients with redundant skin in the anterior neck, it makes an ideal flap, leaving a well-hidden donor site.

## Conclusions

CGCG in its aggressive form can cause extensive bony destruction thereby leading to cosmetic deformities as well as functional impairments; therefore, elimination of such lesions with adequate safety margins is of paramount importance in order to prevent any future chances of recurrence. In our case, we saw a similar presentation of an aggressive lesion at an uncommon age involving the posterior mandibular region. Considering the nature of the lesion along with patient age, we managed the case in an evidence-based manner by extended marginal mandibulectomy and bone debridement. Since such a procedure leaves a weakened bone as well as a large defect, we used a titanium plate to strengthen the mandible and a submental island flap to reconstruct the large surgical defect. The outcome was satisfactory with no recurrence after one year.
